# Memristors for Neuromorphic Circuits and Artificial Intelligence Applications

**DOI:** 10.3390/ma13040938

**Published:** 2020-02-20

**Authors:** Enrique Miranda, Jordi Suñé

**Affiliations:** Departament d’Enginyeria Electrònica, Universitat Autònoma de Barcelona, 08193 Bellaterra, Spain; enrique.miranda@uab.cat

**Keywords:** artificial intelligence, neural networks, resistive switching, memristive devices, deep learning networks, spiking neural networks, electronic synapses, crossbar array, pattern recognition

## Abstract

Artificial Intelligence has found many applications in the last decade due to increased computing power. Artificial Neural Networks are inspired in the brain structure and consist in the interconnection of artificial neurons through artificial synapses in the so-called Deep Neural Networks (DNNs). Training these systems requires huge amounts of data and, after the network is trained, it can recognize unforeseen data and provide useful information. As far as the training is concerned, we can distinguish between supervised and unsupervised learning. The former requires labelled data and is based on the iterative minimization of the output error using the stochastic gradient descent method followed by the recalculation of the strength of the synaptic connections (weights) with the backpropagation algorithm. On the other hand, unsupervised learning does not require data labeling and it is not based on explicit output error minimization. Conventional ANNs can function with supervised learning algorithms (perceptrons, multi-layer perceptrons, convolutional networks, etc.) but also with unsupervised learning rules (Kohonen networks, self-organizing maps, etc.). Besides, another type of neural networks are the so-called Spiking Neural Networks (SNNs) in which learning takes place through the superposition of voltage spikes launched by the neurons. Their behavior is much closer to the brain functioning mechanisms they can be used with supervised and unsupervised learning rules. Since learning and inference is based on short voltage spikes, energy efficiency improves substantially. Up to this moment, all these ANNs (spiking and conventional) have been implemented as software tools running on conventional computing units based on the von Neumann architecture. However, this approach reaches important limits due to the required computing power, physical size and energy consumption. This is particularly true for applications at the edge of the internet. Thus, there is an increasing interest in developing AI tools directly implemented in hardware for this type of applications. The first hardware demonstrations have been based on Complementary Metal-Oxide-Semiconductor (CMOS) circuits and specific communication protocols. However, to further increase training speed andenergy efficiency while reducing the system size, the combination of CMOS neuron circuits with memristor synapses is now being explored. It has also been pointed out that the short time non-volatility of some memristors may even allow fabricating purely memristive ANNs. The memristor is a new device (first demonstrated in solid-state in 2008) which behaves as a resistor with memory and which has been shown to have potentiation and depression properties similar to those of biological synapses. In this Special Issue, we explore the state of the art of neuromorphic circuits implementing neural networks with memristors for AI applications.

## 1. Introduction

The applications of Artificial Intelligence (AI) and their impact on global society are currently growing at an exponential pace. Image recognition, speech processing, business management and optimization, stock markets, medical diagnosis, global climate forecast, autonomous cars, and science discovery are only some of the present AI applications. The term AI was coined back in the late fifties, but it is only in the last decade that, due to the impressive improvements in computing power driven by the Moore’s law, AI has been successfully applied in many areas, even overcoming humans in some tasks [1]. At this point, it is convenient to distinguish between conventional (narrow) AI applications, which are designed for one specific task, and Artificial General Intelligence (AGI) programs that aim at emulating human in the most general situations. All big players such as IBM, Google, Facebook, Microsoft, Baidu and practically everybody who’s anybody in the AI field is committed to achieve AGI. The main idea behind AGI research programs is that once you solve intelligence, you can use it to solve everything else. AI is heralded as a revolutionary technology for the 21st century, it has many applications for the good but, in its AGI version, it has also been signaled as one of the significant risks for the future of humanity [2].

### 1.1. Artificial Intelligence and Its Implementation in Software

The internet has fueled AI applications providing huge amounts of data which can be fed into artificial neural networks (ANNs) to reveal relevant information about complex systems which could not otherwise be analyzed. ANNs are inspired in the low-level structure of the brain and are formed by artificial neurons interconnected by artificial synapses exhibiting plasticity effects. During the training of an ANN, large amounts of data are provided through the input neurons and the strength of the synaptic interconnections are progressively modified until the network learns how to classify not only the training data but also unforeseen data of a similar kind. The process of data recognition of the trained network is most often called inference. In the training phase, we can distinguish between supervised and unsupervised learning algorithms. Supervised learning requires the labelling of the raw data while unsupervised learning can directly deal with unstructured data. Supervised learning is implemented with the so-called Deep Neural Networks (DNNs) but also with other types of ANNs DNNs consist in an ordered arrangement of neuron layers interconnected with the adjacent layers by synapses. There is an input layer through which the data are supplied, an output layer which provides the processed information and, one or several hidden layers with different hierarchical levels of data representation. The actual architecture of interconnections gives rise to different types of networks optimized for different applications (fully connected networks, convolutional networks, recurrent networks, etc.). DNNs are trained using the backpropagation algorithm which consists in calculating an error function (the cost function) as the sum of the squared differences between the obtained and the expected outputs for mini batches (a subset of whole training database, which are called epochs. The cost function is progressively minimized using the stochastic gradient descent method and the back propagation of errors from output to input allows modifying the synaptic weights and train the system. In unsupervised learning networks, the expected response is not a priori known and hence, no cost function can be minimized and the backpropagation technique cannot be used. These networks learn to classify the data by themselves and the meaning of the classification results has to be finally interpreted. These systems are very promising to reveal previously unnoticed features in unstructured data and have the advantage of not requiring data labelling. For a nice recent review of DNNs characteristics and applications, see the work of Hinton [1].

Another type of networks are the so-called Spiking Neural Networks (SNN), in which the data moves back and forward through the network in the form of spikes generated by the neurons. These spikes progressively modify the synaptic weights in a way which is much more similar to the way synapses are potentiated or depressed in the human brain. In general, these systems are energetically much more efficient and use bioinspired learning rules. One example of these rules is the so-called Spike Time-Dependent Plasticity (STDP) in which a synapsis is potentiated or depressed when forward and backwards voltage spikes overlap at the terminals of the device. When the forward pulse arrives before than the backwards pulse (stimulus-response causal relation), the synapsis is potentiated, while if the backwards pulse arrives first, the synapsis is depressed. The STPD learning rules is very typically and easily applied for unsupervised learning in SNNs. However, there are also many works developing supervised learning algorithms to SNN like, for example, the spiking spatio-temporal back-propagation technique. The overwhelming success of DNNs has somehow slowed down the progress of SNNs applicationsHowever, these are certainly the most promising systems for the future, including applications which continue learning along their operation live. Nice reviews about SNNs can be found in the works of Brette et al. [3] and Tavanaei et al. [4].

Most AI algorithms have been implemented by software programs which run on conventional computing systems with a Von Neumann architecture such as central processing units (CPUs), graphical processing units (GPUs) and field programmable gate arrays (FPGAs). Recently, especially designed application specific integrated circuits such as the tensor processing unit (TPU) have been introduced to optimize the type of operations required for training and inference. In all these computing systems, the memory and processing units are physically separated so that significant amounts of data need to be shuttled back and forth during computation. This creates a performance bottleneck both in processing speed due to the memory latency and in power consumption due to the energy requirements for data retrieving, transportation and storage. It must be noticed that most of the involved energy is related to memory operations which can consume up to more than 1000X the energy consumed in arithmetic operations. [5] The problem is aggravated by the fact that deep learning systems are growing significantly in size (more and more hidden layers) in order to improve output accuracy and, as a consequence, training time, cost and energy consumption significantly increase. This growth of size has also the drawback that it is difficult to distribute large models through over-the-air update for applications at the edge of the internet, such as autonomous cars or mobile phone applications. As for the size increase and the associated reduction of training speed, we can consider the evolution of Microsoft ResNet system for image recognition. ResNet18 (18 layers of neurons) required 2.5 days of training to reach an error rate of about 10.8% while ResNet152 training takes 1.5 weeks to reach a prediction failure rate of 6.2%. Let us consider the case of AlphaGo as a last example. In 2016, AlphaGo, a complex AI tool developed by DeepMing (Google), defeated the top-ranking professional player, Lee Sedol, in the ancient game of Go which, according to the developers is 10^100^ times more complex than chess [6]. AlphaGo used deep neural networks trained by a combination of supervised learning from human expert games, and reinforcement learning, based on a reward for success, a way of learning inspired in phycology. The two neural networks of AlphaGo used Monte Carlo tree search programs to simulate thousands of random games of self-play [6]. It its largest distributed version, running on multiple machines, used 40 search threads, 1920 CPUs and 280 GPUs. On the other hand, while Lee Sedol consumed about 20 Watts of power to play, AlphaGo power consumption was of approximately 1 MW (200 W per CPU and 200 W per GPU), i.e. an electricity bill of USD 300 was genenerated for a single game. Given the required complexity of computing resources and huge amounts of energy consumption, alternative approaches to the implementation of AI tools are required. In this regard, the hardware implementation of AI and, in particular, neural networks built with memristors, might be the next step in the way towards reduced size, energy efficient AI systems with performace much closer to that of the human brain.

### 1.2. Artificial Intelligence and Its Hardware Implementation 

Nowadays, there is a rising interest for the hardware implementation of ANNs using neuromorphic circuits. These are ordered networks of electron devices implementing artificial neurons and their synaptic interconnections. These hardware networks allow in-memory computing (computation performed directly on a non-volatile memory array), massive parallelism and huge improvements in power consumption. Moreover, they are highly suited for applications at the edge of the internet.

The first hardware implementations of neural networks are those based on CMOS technology. In these systems, neurons are based on CMOS circuits (both for computing and memory) and the required high density of interconnections is usually implemented by a virtual wiring system consisting in a digital bus and a special purpose communication protocol. Using this approach, large-scale SNN chips have been developed, reaching up to one million neurons per chip [7]. Interconnecting these chips on a board or a wafer and assembling them to form more complex systems have also been demonstrated. This approach is scalable to implement very complex neuromorphic systems. However, these systems require large amounts of circuitry and are costly in terms of area and energy consumption. Scaling these systems to the complexity of the brain (roughly 10^11^ neurons and 10^15^ synapses) would require a space of the size of a room. These drawbacks have motivated the exploration of other hardware alternatives such as those which combine CMOS for neuron implementation and memristors for synapses. This is the scope of this special issue namely, the application of memristors to build improved neuromorphic systems for AI.

The solid-state nanoelectronic implementation of the memristor was reported for the first time in 2008 by the HP group led by Stanley Williams [8]. In 1971, Leon Chua used symmetry arguments to predict a device which should relate electric charge and magnetic flux [9], just as inductors relate current and magnetic flux or capacitors relate charge and voltage. However, the memristor is better understood as a resistor with memory. A resistor whose value depends on an internal variable that changes with the applied voltages or currents and hence, it is able to store information in an analogue and non-volatile manner. On the other hand, these devices (with can be scaled down to 10 nm) can be fabricated in dense crossbar arrays (an array of perpendicular wires with a memristor at each crossing point) in the back-end of the CMOS process. Moreover, these layers can be stacked one on top of another to provide a highly dense 3D array of non-volatile memory (an implementation of the so-called storage-class memory) or, alternatively, an array of interconnections with synaptic properties for neuromorphic circuits. These memory arrays allow toperform computing tasks within the data themselves using for example their capability to perform operations such as matrix-vector multiplication (MVM) in an analogue, highly parallel and energy-efficient way. This type of one-step MVM calculations are based on physical laws such as the Ohm’s law and the Kirchoff´s law and they are the basis of in-memory computing. This is a very important change of paradigm which overcomes the limitations of the Von Neumann architecture for some tasks. On the other hand, these hybrid CMOS/memristor based neuromorphic systems will allow reducing the energy consumption by a factor of at least 10^3^ with respect to pure CMOS implementations. Furthermore, there is a very relevant reduction of area so that a complex neural system with the density of the brain neurons and synapses could in principle be fabricated in a single board. The possibility of fabricating memristors with short-term non-volatility also points out to the possibility of implementing purely memristive DNNs and SNNs [10]. By purely memristive we refer to systems that implement both synapses and neurons with memristors.

In recent years, many different device concepts have been considered for implementing the memristor. These include the phase change memory (PCM), in which resistance is modified by partial crystallization/amorphization of a small volume of chalcogenide material; the Resistive Random Access Memory (RRAM) where conductance change is related to the electro-ionic modulation of a conducting filament across an oxide layer (mainly in binary oxides such as Ta_2_O_5_, Al_2_O_3_, HfO_2_, TiO_2_, …) or to the homogenous modulation of an injection barrier (mainly in complex perovskite oxides); spintronic devices in which the memristor internal variable is the partial spin polarization, ferroelectric devices which use changes in the dielectric polarization to modify the device conductance, and others. Memristors have been implemented mainly in two-terminal devices (2T) but recently, three-terminal (3T) structures are also being explored to optimize some fundamental device properties for neuromorphic applications such as linearity in the conductance change and conduction symmetry.

Recently, there have been several hardware demonstrations of neural networks with synaptic interconnections implemented with memristors. The very first demonstration was that of Prezioso and coworkers [11] who implemented a single layer perceptron using a 12 × 12 passive crossbar array of RRAM. Using supervised training, they demonstrated classification of a small dataset of 3x3 images into three classes. Recently, the same research group presented another demonstrator of a perceptron classifier with one hidden layer implemented with two purely passive 20 × 20 crossbar arrays board-integrated with discrete conventional components [12]. With this system, they reached an error rate of only 3% with ex-situ supervised learning. Larger arrays have also been recently reported but all these incorporate an MOS transistor (selector) in series with the memristor (1T1R configuration) at each cross point. The transistor increases the required area and compromises the desired scalability. However, it is necessary to limit the current thus avoiding damaging the memristor during the forming/potentiation phases. On the other hand, the transistor allows to eliminate the crosstalk effects (sneak-path problem) which are increasingly significant for large synaptic arrays. With this 1T1R structure, a hardware accelerator based on 165,000 PCM synapses was implemented and used for image classification using the Modified National Institute of Standards and Technology (MNIST) database of handwritten digits [13]. The same set of MNIST data was also recently used for the in-situ supervised training of 1T1R RRAM synaptic crossbars of about 8000 memristors, showing high accuracy and tolerance to defective devices (stuck at low conductance) and reaching high recognition accuracy [14]. Also remarkable is the recent demonstration of the ex-situ training of a two-layer perceptron DNN implemented with a 4Kbit 1T1R RRAM array which not only achieved high accuracy but also very low power consumption [15].

Progress in the implementation of neural networks using memristors as synapses is remarkable. However, many issues still need to be resolved both at the material, device and system levels so as to simultaneously achieve high accuracy, low variability, high speed, energy efficiency, small area, low cost, and good reliability. This requires combined research efforts in these three interconnected areas: devices, circuits and systems. Towards this goal, high quality compact behavioral models are a very important ingredient to explore compare different devices at the circuit and system levels. In this regard, Stanley Williams recently made a call to the memristor research community requesting high quality compact models for the circuit designers and systems architects to use with confidence for their circuit and system simulations and validations [16].

## 2. Synopsis

In this special issue we are honored to have two invited review papers by recognized leaders in the field. Camuñas-Mesa, Linares-Barranco and Serrano-Gotarredona focus their review on the implementation of SNNs with hybrid memristor-CMOS hardware, and review the basics of neuromorphic computing and its CMOS implementation [17]. Milo, Malavena, Monzio Compagnoli and Ielmini, mainly focus on memristive devices implementing electronic synapses in neuromorphic circuits [18]. They consider different memory technologies for brain-inspired systems including mainstream flash memory technologies, and memristive technologies with 2T and 3T structures. Finally, they review recent results on the use of these devices in both SNN and DNN memristive/CMOS neuromorphic networks. Both reviews provide an updated complementary view of the state-of-the-art in the implementation of neuromorphic systems with memristive synapses. Besides these two featured papers, a total number of 11 contributed papers complete this special issue.

Truong proposes a method to correct the line resistances when writing the desired values of conductance in DNN for feedforward applications [19]. Circuit simulations of a 64 × 16 single layer perceptron for the recognition of 26 characters (8 × 8 grayscale pixels) support significant improvements of network recognition when line resistance increases above 1.5Ω.

Wang et al. report an optimized RRAM device with a forming-free Al_2_O_3_/TaO_X_ stacked oxide which shows non-filamentary switching, and an analog bipolar switching that permits to program the conductance in an ANN with a high precision (error rate < 10%) [20]. The device shows relevant synaptic properties such as long-term potentiation and depression, spike-time dependent plasticity and pulse-pair facilitation. Although the conductance change of such synapses as a function of the number of constant voltage amplitude pulses is highly nonlinear, optimization of the training method allows obtaining rather linear changes and this would allow good accuracy in ANNs.

Van Nguyen et al. contribute to this issue with two papers that deal with mimicking the brain’s neocortical operation in hardware. In the first one, they propose a memristor-CMOS hybrid circuit for the temporal-pooling of sensory and hippocampal information [21]. This circuit is composed by an input layer which combines sensory and temporal/location information in a memristor crossbar. The output layer of neurons also contains a memristor crossbar and integrates the information to make predictions. Both input and output layers contain memristor crossbars and standard CMOS circuits such as current-voltage converters, comparators, AND gates, latches and other circuits. Instead of the backpropagation algorithm, they use the much simpler Hebbian learning, which can be suitable for online learning. Moreover, the authors verify their proposal by circuit simulation with a Verilog-A phenomenological model for the memristor. Application of the circuit to the Enhanced-MNIST database demonstrates very good accuracy in both word and sentence recognition. In their second paper, they deal with reducing the effects of defects in the memristor crossbars such a stuck-at faults and memristor variations [22]. First, they show that the boost-factor adjustment can make the system fault-tolerant by suppressing the activation of defective columns. Second, they propose a memristor-CMOS hybrid circuit with the boost-factor adjustment to realize a defect-tolerant Spatial Pooler in hardware. Using the MNIST database, they show that the recognition accuracy is reduced only by 0.6% by the presence of up to 10% crossbar defects, with a very low energy overhead related to boost factor adjustment.

Fernández-Rodríguez et al. deal with a new class of 3T memristive devices based on the Metal-Insulator-Transition (MIT) in YBa_2_Cu_3_O_7-δ_ (YBCO), a complex perovskite oxide with well-known high-T superconducting properties [23]. At 300K, YBCO doesn’t show any sign of superconduction but, small changes in the oxygen concentration produce large changes in resistance due to the MIT so that reversible non-volatile memory effects are observed. The authors fabricate prototype 3T transistor-like devices (memistors) which allow demonstrating volume switching effects different from the widely studied filamentary or interfacial effects. The reported results allow the fabrication of highly functional trans-synaptic devices where the input signal modifies the conductance of the output channel.

Rodríguez et al. investigate the origin of novel laser-fabricated graphene oxide memristors [24]. They use numerical tools linked to Time Series Statistical Analysis to reveal that these memristors are based on a local change of the stoichiometry in a conducting filament (as it is the case in most of binary oxides memristor). For the filament conduction they use the widely known point-contact model.

Hajtó et al. deal with the problem of the high variability of memristor properties [25]. First, they thoroughly discuss the need of more reliable devices for ANNs and neuromorphic in-memory computation, which require multi-state digital memristors and analog memristors, respectively. To reduce variability, they propose to use several interconnected memristors (memristor emulator circuit) at each synaptic location. After having simulated these circuits in previous works, in this issue they present real measurements demonstrating the change of operation properties of the emulator circuits and the reduction of the variability index. The evident drawbacks of this approach are the increase of effective consumed chip area (either in 2D crossbars or 3D stacks of crossbars) and the reduction of energy efficiency.

Pedró et al. deal with the simulation of fully unsupervised learning in self-organized Kohonen networks [26]. After experimentally characterizing HfO_2_ memristors and demonstrating STDP synaptic properties in these devices, they propose a set of waveforms to minimize conductance change non-linearity. Using a realistic compact behavioral model for these memristors, they simulated the neuromorphic system, thus testing the learning algorithm. They also discuss that the selected system design and learning scheme permits to concatenate multiple neuron layers for autonomous hierarchical computing.

The high complexity and limited knowledge about the physical processes taking place in RRAM memristors is nowadays hampering the development, optimization and application of these devices. Moreover, these devices are to be used in different applications, such as embedded non-volatile memories, SNNs and DNNs, and the device requirements change for each application. Thus, La Torraca et al. present a multi-scale simulation platform which includes all physical mechanisms such as charge transport, charge trapping, ion generation, diffusion, drift and recombination in an environment that considers the 3D distribution of temperature and electric field [27]. This multiscale approach allows simulating the performance of RRAM devices connecting their electrical properties to the underlying microscopic mechanisms, optimizing their analog switching performance as synapses, determining the role of electroforming and studying variability and reliability. Using this platform, the device performance can be optimized for different applications with different RRAM requirements.

Sun et al. discuss the application of 3D crossbar structures for the implementation of multi-layer neuromorphic networks (DNNs) [28]. They focus on RRAM memristors with a 3D structure and propose a new optimization method for machine learning weight changes that considers the properties of Vertical Resistive Random Access Memories (VRRAM). The operating principle of VRRAM allows to simplify the structure of the neuron circuit. The studied devices are promising for high-density neural network implementations.

Cisternas Ferri et al. use a phenomenological compact model for RRAM memristors, already experimentally validated in previous works, to construct a memristor emulator [29]. The main advantage of emulators over simulators is that they can be connected to external circuits to characterize their behavior in realistic environments. Moreover, the parameters of the emulated device can be arbitrarily changed so as to optimize the circuit performance and guide the ulterior fabrication of devices with optimum properties for a certain application. The memristor model is implemented using an Arduino microprocessor which solves the required differential equations. An analog-to-digital converter in the microcontroller measures the voltage on a digital potentiometer and the microprocessor changes its resistance accordingly. The emulator is validated comparing the obtained experimental results with model simulations (sinusoidal frequency memristive response, STDP, and response to voltage pulses). Finally, the emulator is introduced in a simple neuromorphic circuit that exhibits the main characteristics of Pavlovian conditioned learning.
